# Protective Effect of *Pyropia yezoensis* Peptide on Dexamethasone-Induced Myotube Atrophy in C2C12 Myotubes

**DOI:** 10.3390/md17050284

**Published:** 2019-05-11

**Authors:** Min-Kyeong Lee, Jeong-Wook Choi, Youn Hee Choi, Taek-Jeong Nam

**Affiliations:** 1 Institute of Fisheries Sciences, Pukyong National University, Busan 46041, Korea; 3633234@hanmail.net (M.-K.L.); wook8309@naver.com (J.-W.C.); 2 Department of Marine Bio-Materials & Aquaculture, Pukyong National University, Busan 48513, Korea; 3 Department of Food Science and Nutrition, Pukyong National University, Busan 48513, Korea

**Keywords:** dexamethasone, myotube atrophy, protein synthesis, proteolytic system, *Pyropia yezoensis* peptide, PYP15

## Abstract

Dexamethasone (DEX), a synthetic glucocorticoid, causes skeletal muscle atrophy. This study examined the protective effects of *Pyropia yezoensis* peptide (PYP15) against DEX-induced myotube atrophy and its association with insulin-like growth factor-I (IGF-I) and the Akt/mammalian target of rapamycin (mTOR)-forkhead box O (FoxO) signaling pathway. To elucidate the molecular mechanisms underlying the effects of PYP15 on DEX-induced myotube atrophy, C2C12 myotubes were treated for 24 h with 100 μM DEX in the presence or absence of 500 ng/mL PYP15. Cell viability assays revealed no PYP15 toxicity in C2C12 myotubes. PYP15 activated the insulin-like growth factor-I receptor (IGF-IR) and Akt-mTORC1 signaling pathway in DEX-induced myotube atrophy. In addition, PYP15 markedly downregulated the nuclear translocation of transcription factors FoxO1 and FoxO3a, and inhibited 20S proteasome activity. Furthermore, PYP15 inhibited the autophagy-lysosomal pathway in DEX-stimulated myotube atrophy. Our findings suggest that PYP15 treatment protected against myotube atrophy by regulating IGF-I and the Akt-mTORC1-FoxO signaling pathway in skeletal muscle. Therefore, PYP15 treatment appears to exert protective effects against skeletal muscle atrophy.

## 1. Introduction

Bioactive peptides are generally composed of between three and 20 amino acid residues, and their activity is based on their amino acid composition and sequence [1]. The short chains of amino acids are in an inactive state within the sequence of the parent protein molecule, but can be liberated by proteolytic enzymes during gastrointestinal digestion or food processing and fermentation processes [2]. These peptides exhibit various physiological activities, such as anti-hypertensive [3], anti-oxidant [4,5,6], and anti-inflammatory effects [7,8] depending on their structural, sequential, and constitutive characteristics.

**Figure 1:**
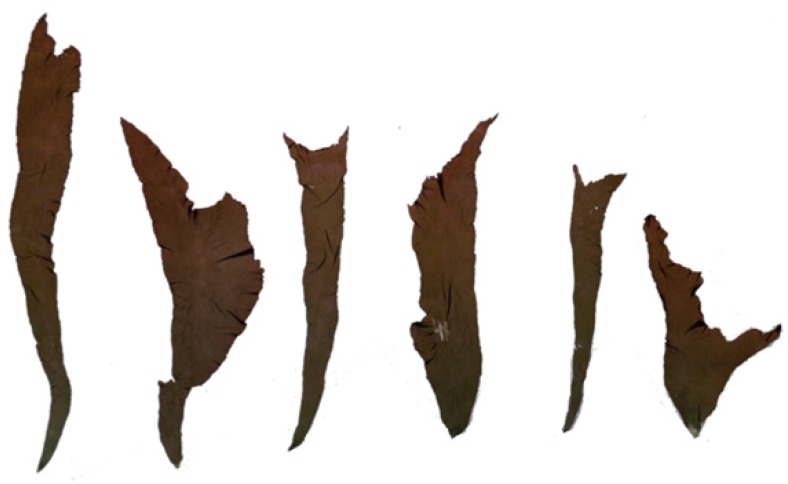
Image of *Pyropia yezoensis* Ueda (Bangiaceae, Rhodophyta).

*Pyropia yezoensis* Ueda (Bangiaceae, Rhodophyta, Figure 1) is a commercially important red seaweed widely used as a food source in Korea, China, and Japan [9]. The protein content of *P. yezoensis* is higher than that of high protein foods such as soybeans, thus providing a rich source of biologically active peptides [10]. *P. yezoensis*-derived peptides are known to exert various biological effects, including antioxidant [11], antitumor [12], and anti-inflammatory activities [13]. Previous studies have reported that peptides synthesized from *P. yezoensis* reduced the inflammatory stress induced by lipopolysaccharides in RAW 264.7 cells, and cytotoxicity induced by acetaminophen in Chang cells [13,14]. Moreover, *P. yezoensis* peptides prevented the endoplasmic reticulum stress induced by perfluorooctane sulfonate in Chang cells [15]. A recent study also demonstrated that *P. yezoensis* peptide promoted collagen synthesis by activating the transforming growth factor-beta (TGF-β)/Smad signaling pathway in human dermal fibroblasts [16]. In addition, *P. yezoensis* peptides also protected against breast cancer by activating the mammalian target of rapamycin (mTOR) signaling pathway in MCF-7 cells [12]. Therefore, *P. yezoensis* is widely used as a health-promoting functional natural material due to its various physiological activities.

Glucocorticoids (GCs) are steroid hormones widely administered for their anti-inflammatory and immunosuppressive activities [17]. However, sustained high-dose administration of GCs may lead to hyperglycemia, weight loss, osteoporosis, depression, hypertension, and skeletal muscle atrophy [18]. Many previous in vivo and in vitro experiments have suggested that synthetic GC dexamethasone (DEX) induces skeletal muscle atrophy by decreasing the protein synthesis rate and increasing the protein degradation rate [19,20].

The inhibitory effect on protein synthesis seen in GC-induced muscle atrophy occurs through multiple mechanisms. First, the catabolic effect of GCs inhibits the transport of amino acids within muscle, limiting protein synthesis [21]. Second, GCs inhibit the secretion of insulin-like growth factor I (IGF-I), which stimulates the phosphorylation of eIF4E-binding protein 4E-BP1 and p70 ribosomal S6 protein kinase (p70S6K), two factors that play key roles in protein synthesis by controlling the initiation of mRNA translation [20,22]. Third, GCs cause muscle atrophy by blocking myogenesis through the inhibition of myogenin, a transcription factor essential for the differentiation of satellite cells into muscle fibers [23]. It is well known that the inhibition of protein synthesis by GCs is mainly due to activation of the IGF-I-Akt-mTORC1 signaling pathway, which is involved in the phosphorylation of 4E-BP1 and p70S6K [24]. Previous studies have demonstrated that GCs in L6 myoblasts decrease the protein levels of insulin receptor substrate-1 (IRS-1), the first upstream component of the Akt-mTOR cascade [25,26]. In addition, GCs have been shown to inhibit the Akt-mTOR pathway by promoting the expression of microRNA miR1 [27]. These observations suggest that excess GCs may cause muscle atrophy by inhibiting the IGF-I-Akt-mTORC1 signaling pathway.

Akt also regulates the ubiquitin-proteasome system and autophagy-lysosomal system via the forkhead box O (FoxO) transcription factors, which are produced in muscle cell catabolism caused by GCs [28,29]. Mammalian cells express three FoxO isoforms, FoxO1, FoxO3, and FoxO4, which have been implicated in the regulation of genes involved in cell death, cell cycle arrest, and metabolism [30]. The absence of growth or survival signals inactivates Akt, thereby attenuating its inhibitory effects on FoxO transcription factors, which permits their translocation from the cytoplasm to the nucleus [31]. Nuclear translocation and activation of FoxO transcription factors are required to upregulate atrogenes, such as atrogin-1/muscle atrophy F-box (MAFbx), muscle RING finger 1 (MuRF1), and cathepsin-L. FoxO3 transfection of skeletal muscle cells was found to be sufficient for upregulating atrogin-1/MAFbx expression and muscle atrophy [32]. Moreover, previous studies employing inhibitors of the different proteolytic pathways have demonstrated that GCs stimulate not only ubiquitin-proteasome system-dependent proteolysis, but also autophagy-lysosomal system-dependent protein breakdown [30]. The role of the autophagy-lysosomal system in muscle atrophy induction by GCs is also demonstrated by the upregulation of muscle cathepsin-L expression [33,34,35] and by the upregulated conversion of LC3-I to LC3-II, an indicator of autophagy [36], in animals administered GCs. These findings suggest that activation of the ubiquitin-proteasome and autophagy-lysosomal systems in skeletal muscle atrophy induced by GC exposure may be mediated by the activation of FoxO through the inhibition of Akt expression.

Our previous study provided molecular evidence that the protective effects of the *P. yezoensis* peptide on DEX-induced muscle atrophy were due to downregulation of the muscle-specific E3 ubiquitin ligases atrogin-1/MAFbx and MuRF1 [37]. The present study was undertaken to investigate whether the protective effects of *P. yezoensis* peptide (PYP15) against DEX-induced myotube atrophy are associated with the proteolytic system, and whether this is regulated by the IGF-I-mediated Akt-mTOR and Akt-FoxO signaling pathways.

## 2. Results

### 2.1. Effects of DEX and PYP15 on C2C12 Myotube Viability

**Figure 2:**
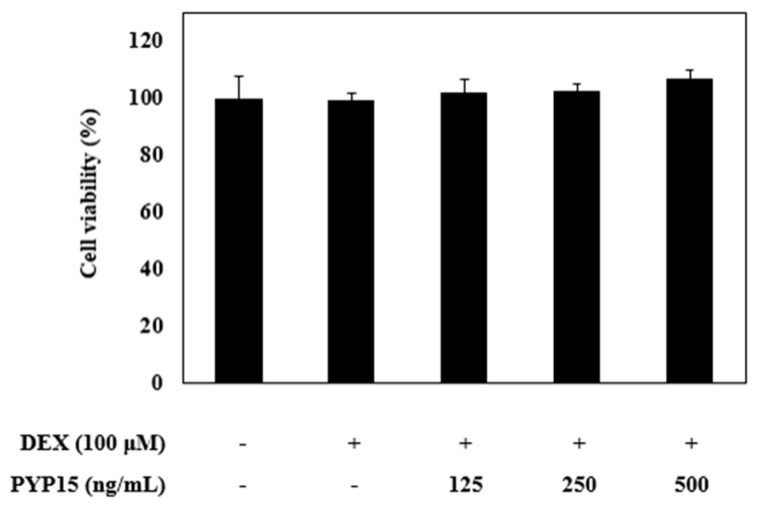
Effects of dexamethasone (DEX) and *Pyropia yezoensis* peptide (PYP15) on the cytotoxicity of C2C12 myotubes. C2C12 myoblasts were seeded in 96-well plates at a density of 1.5 × 10^4^ cells/well and were allowed to attach for 24 h. After differentiation, the cells were treated with 100 μM DEX and 500 ng/mL PYP15 for 24 h. The viability of C2C12 myotubes was measured by MTS assay. The values are the mean ± SDs of three independent experiments.

To evaluate the cytotoxic effects of PYP15 on C2C12 myotubes, MTS [(3-4,5-dimethylthiazol-2-yl)-5-(3-carboxymethoxyphenyl)-2-(4-sulfonyl)-2H-tetrazolium)] assays were carried out. C2C12 myotubes were incubated for 24 h with 100 μM DEX and PYP15 at concentrations ranging from 0 to 500 ng/mL. The DEX concentration (100 μM) was determined in a previous study [37]. As shown in Figure 2, PYP15 did not affect cell viability up to a concentration of 500 ng/mL. Thus, all subsequent experiments were performed 24 h after treatment with 500 ng/mL PYP15, which is the appropriate concentration that does not induce cytotoxicity, as described by Choi et al. [38].

### 2.2. PYP15 Treatment Attenuates the DEX-Induced Reduction in Insulin-Like Growth Factor I Receptor (IGF-IR) and IRS-1 Phosphorylation in C2C12 Myotubes

**Figure 3:**
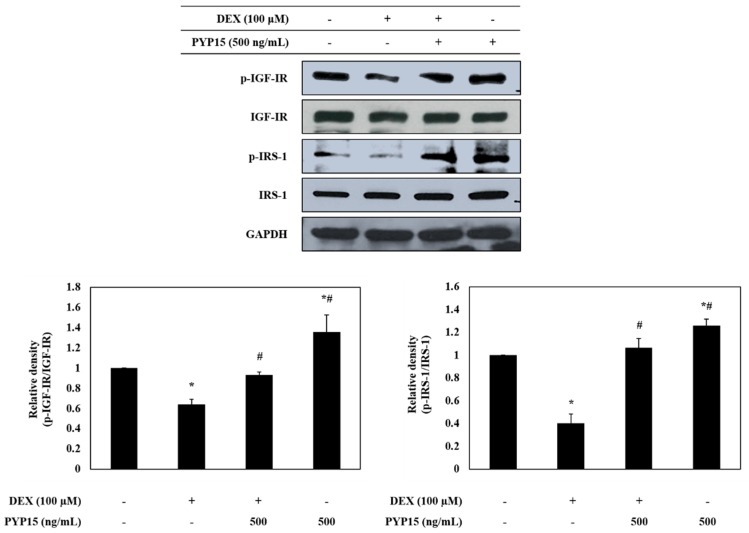
Effects of PYP15 on the phosphorylation of insulin-like growth factor I receptor (IGF-IR) and insulin receptor substrate 1 (IRS-1) in DEX-treated C2C12 myotubes. C2C12 myotubes were treated for 24 h with 100 μM DEX in the absence or presence of 500 ng/mL PYP15. The protein levels for p-IGF-IR, IGF-IR, p-IRS-1, and IRS-1 were assessed as described in Section 4. Glyceraldehyde-3-phosphate dehydrogenase (GAPDH) was the loading control. The values are the mean ± SD of three independent experiments. * P < 0.05 vs. corresponding control; *# P* < 0.05 vs. corresponding only DEX treatment.

To determine whether PYP15 treatment led to changes in the IGF-I pathway in DEX-treated C2C12 myotubes, phosphorylation levels of insulin-like growth factor I receptor (IGF-IR) and insulin receptor substrate 1 (IRS-1) were examined using Western blot analysis. As shown in Figure 3, p-IGF-IR and p-IRS-1 protein expression levels was markedly decreased in DEX-stimulated C2C12 myotubes. However, the DEX-induced downregulation of p-IGF-IR and p-IRS-1 was attenuated by 500 ng/mL PYP15 treatment. Furthermore, C2C12 myotubes treated with PYP15 alone exhibited marked upregulation of the p-IGF-IR and p-IRS-1 protein expression levels compared with untreated control cells. These results suggest that PYP15 could induce muscle hypertrophy through activation of IGF-I signaling.

### 2.3. PYP15 Treatment Attenuates the DEX-Induced Downregulation of the Akt-mTORC1 Signaling Pathway in C2C12 Myotubes

**Figure 4:**
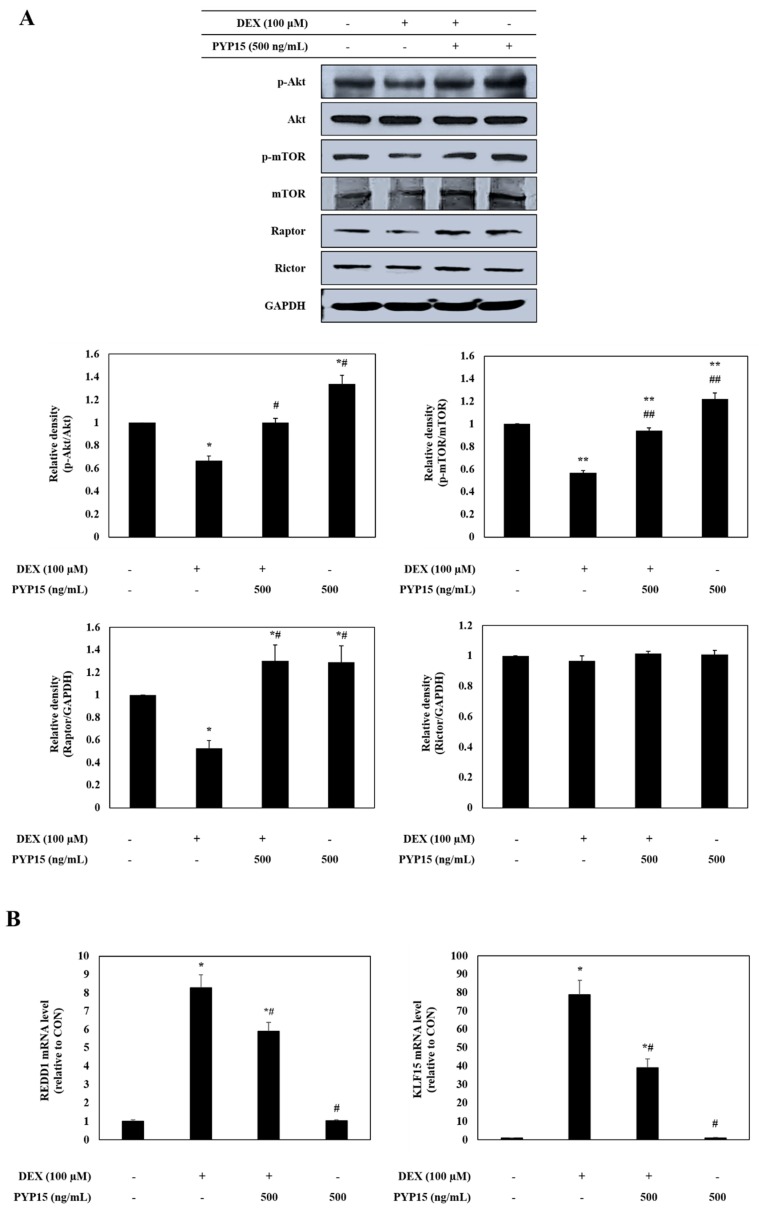

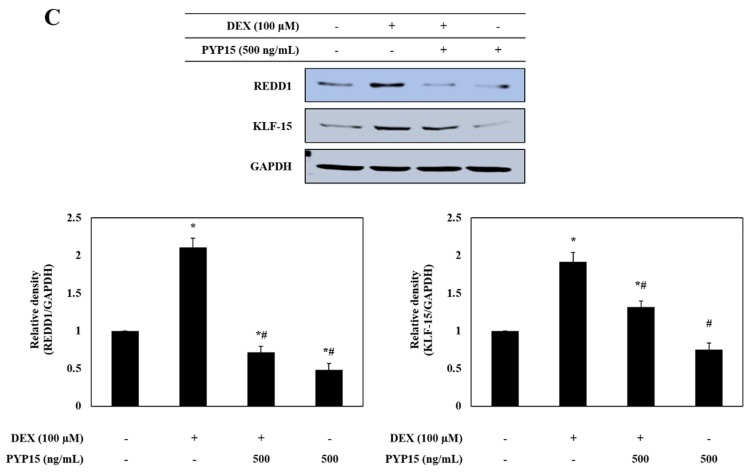
Effects of PYP15 on the Akt/mammalian target of rapamycin (mTOR) signaling pathway in DEX-stimulated C2C12 myotubes. C2C12 myotubes were treated for 24 h with 100 μM DEX in the absence or presence of 500 ng/mL PYP15. (**A**) The protein levels for p-Akt, Akt, p-mTOR, mTOR, Raptor, and Rictor were assessed as described in Section 4. (**B**) The mRNA levels for REDD1 and KLF15 were assessed as described in Section 4. (**C**) The protein levels for REDD1 and KLF15 were assessed as described in Section 4. GAPDH was the loading control. The values are the mean ± SD of three independent experiments. * P < 0.05 vs. corresponding control; *# P* < 0.05 vs. corresponding only DEX treatment.

To further investigate the downstream signals regulated by activation of IGF-I signaling, the protein levels of Akt-mTORC1 pathway members were measured in C2C12 myotubes. As shown in Figure 4A, p-Akt and p-mTOR protein expression levels were markedly decreased in DEX-stimulated C2C12 myotubes. However, the DEX-induced downregulation of p-Akt and p-mTOR was attenuated by 500 ng/mL PYP15 treatment. In addition, to determine whether PYP15 induced changes in mTORC1 and mTORC2 protein levels in DEX-induced myotube atrophy, the protein and mRNA levels of Raptor, Rictor, REDD1, and KLF-15 were examined. As expected, Raptor protein expression levels was markedly decreased in DEX-stimulated C2C12 myotubes. However, the DEX-induced downregulation of Raptor was attenuated by 500 ng/mL PYP15 treatment (Figure 4A). The DEX-induced upregulation of REDD1 and KLF-15 was attenuated by 500 ng/mL PYP15 treatment (Figure 4B,C). However, no significant differences were observed in Rictor levels compared with the control group (Figure 4A). These results demonstrate that PYP15 exerted protective effects against DEX-induced myotube atrophy via mTORC1 signaling activation.

### 2.4. PYP15 Treatment Attenuates the DEX-Induced Decreases in p70S6K and 4E-BP1 Phosphorylation in C2C12 Myotubes

**Figure 5:**
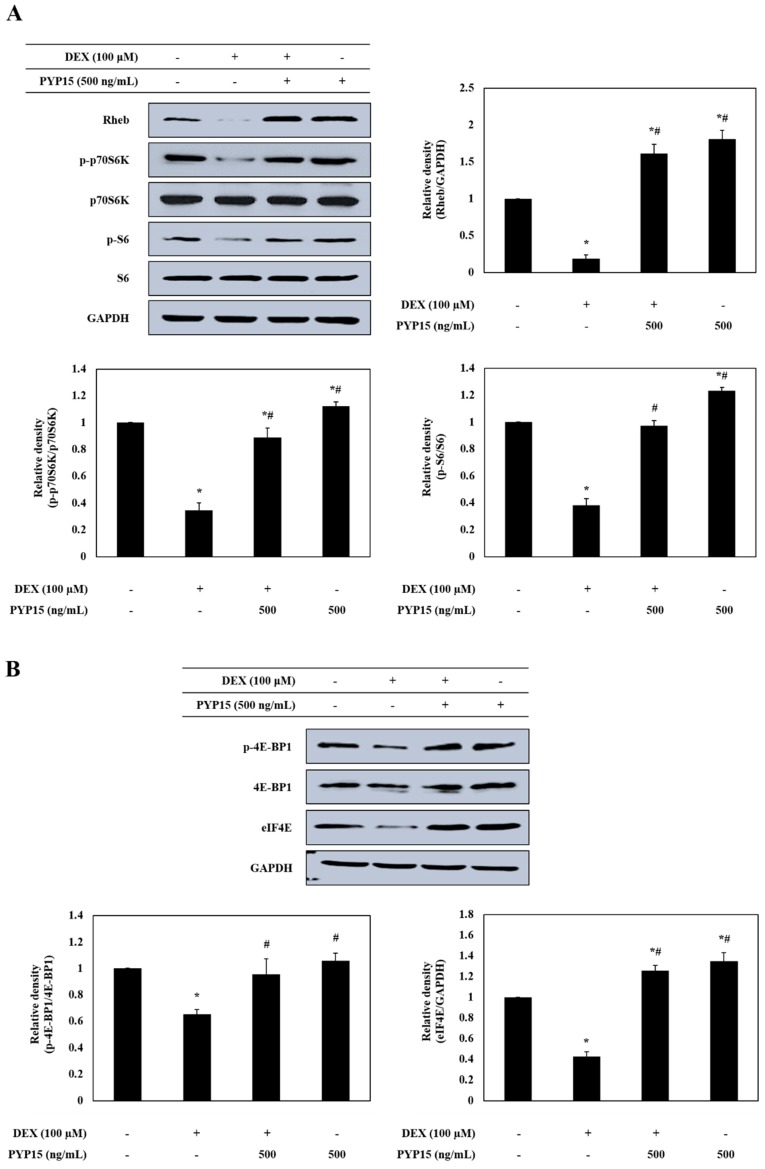
Effects of PYP15 on the mTORC1 downstream signaling components in DEX-stimulated C2C12 myotubes. C2C12 myotubes were treated for 24 h with 100 μM DEX in the absence or presence of 500 ng/mL PYP15. (**A**) The protein levels for Rheb, p-p70S6K, p70S6K, p-S6, and S6 were assessed as described in Section 4. (**B**) The protein levels for p-4EBP1, 4E-BP1, and eIF4E were assessed as described in Section 4. GAPDH was the loading control. The values are the mean ± SD of three independent experiments. * P < 0.05 vs. corresponding control; *# P* < 0.05 vs. corresponding only DEX treatment.

To further determine the downstream signals regulated by mTORC1 activation, the protein levels of p70S6K and 4E-BP1 signaling members were measured in C2C12 myotubes. As shown in Figure 5A, Rheb protein expression levels were markedly decreased in DEX-stimulated C2C12 myotubes. However, the DEX-induced downregulation of Rheb was attenuated by 500 ng/mL PYP15 treatment. In addition, the DEX-induced downregulation of p-p70S6K, p-S6, p-4E-BP1, and eIF4E was attenuated by 500 ng/mL PYP15 treatment (Figure 5A,B).

### 2.5. PYP15 Treatment Downregulates the DEX-Induced Increase in Nuclear Translocation of FoxO1 and FoxO3a in C2C12 Myotubes

**Figure 6:**
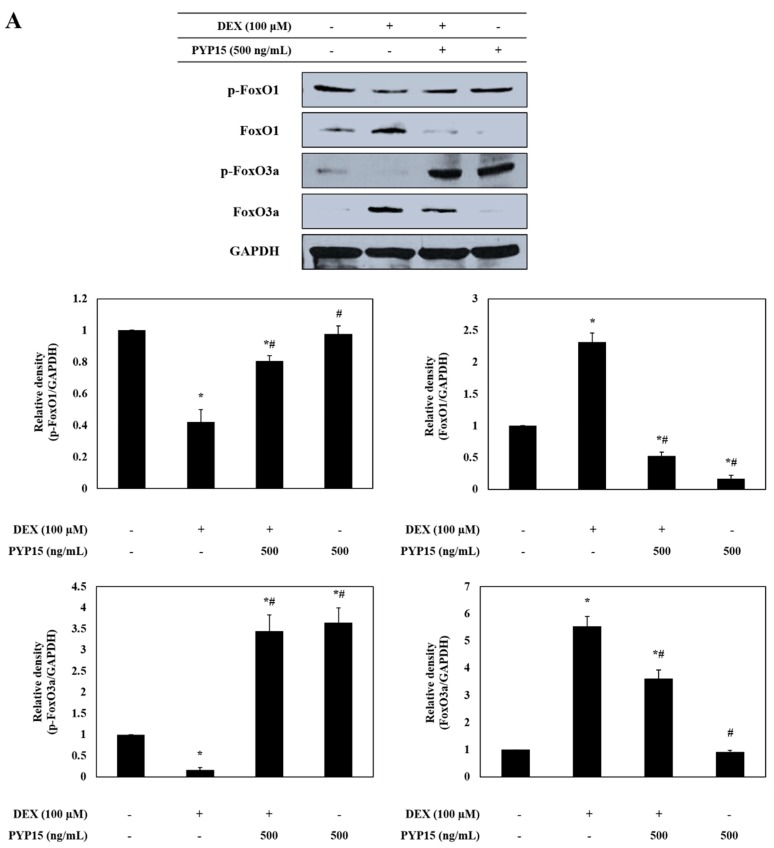

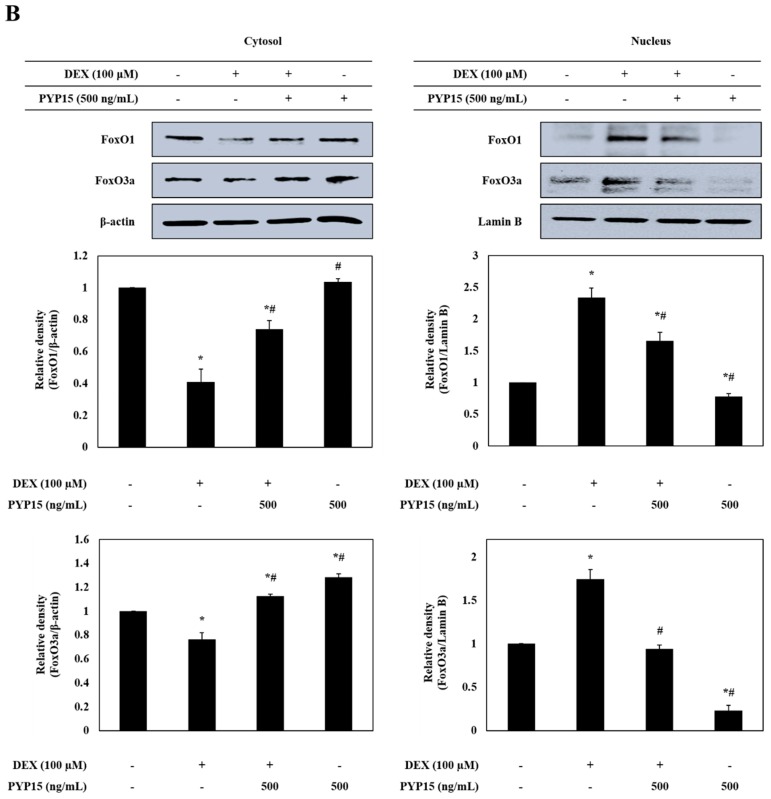
Effects of PYP15 on the activation and translocation of forkhead box O (FoxO) transcription factors FoxO1 and FoxO3a in DEX-stimulated C2C12 myotubes. C2C12 myotubes were treated for 24 h with 100 μM DEX in the absence or presence of 500 ng/mL PYP15. (**A**) The protein levels for total p-FoxO1, FoxO1, p-FoxO3a, and FoxO3a were assessed as described in Section 4. (**B**) The protein levels for cytosolic and nucleus fractions were assessed as described in Section 4. GAPDH, β-actin, and lamin B were the loading control. The values are the mean ± SD of three independent experiments. * P < 0.05 vs. corresponding control; *# P* < 0.05 vs. corresponding only DEX treatment.

To further determine the mechanism underlying transcriptional control by PYP15, the effects of PYP15 on the transcriptional activation of FoxO1 and FoxO3a were measured in DEX-stimulated C2C12 myotubes. As shown in Figure 6A, FoxO1 and FoxO3a protein expression levels were significantly increased in DEX-stimulated C2C12 myotubes (*P* < 0.05). However, the DEX-induced upregulation of total FoxO1 and FoxO3a was attenuated by 500 ng/mL PYP15 treatment. In addition, the DEX-induced reduction in p-FoxO1 and p-FoxO3a was attenuated by 500 ng/mL PYP15 treatment. The dephosphorylation of FoxO1 and FoxO3a accelerated their nuclear translocation. The levels of nuclear FoxO1 and FoxO3a were significantly increased by DEX treatment, but were significantly decreased by PYP15 treatment (*P* < 0.05, Figure 6B).

### 2.6. PYP15 Treatment Inhibits DEX-Induced 20S Proteasome Activity in C2C12 Myotubes

**Figure 7:**
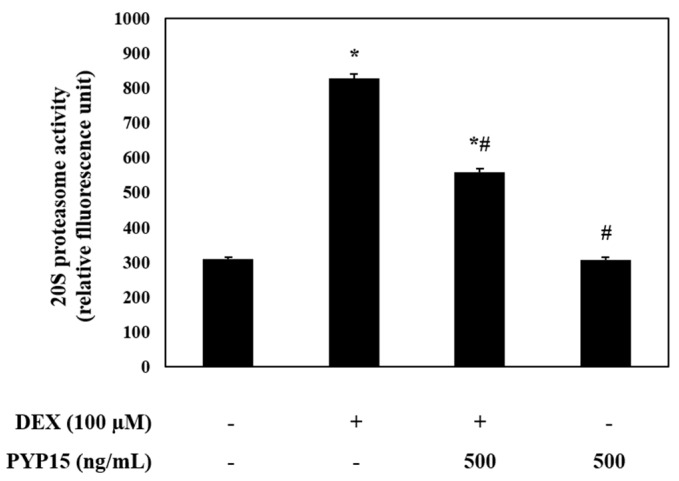
Effects of PYP15 on 20S proteasome activity in DEX-stimulated C2C12 myotubes. 20S proteasome activity was assessed as described in Section 4. The values are the mean ± SD of three independent experiments. * P < 0.05 vs. corresponding control; *# P* < 0.05 vs. corresponding only DEX treatment.

To identify the ubiquitin-proteasome system regulated by FoxO transcription factors, 20S proteasome activity in C2C12 myotubes was measured using an ELISA kit. As shown in Figure 7, DEX treatment significantly increased 20S proteasome activity (*P* < 0.05). However, treatment with PYP15 attenuated the DEX-induced increase in 20S proteasome activity.

### 2.7. PYP15 Treatment Downregulates the DEX-Induced Activation of the Autophagy-Lysosomal System in C2C12 Myotubes

**Figure 8:**
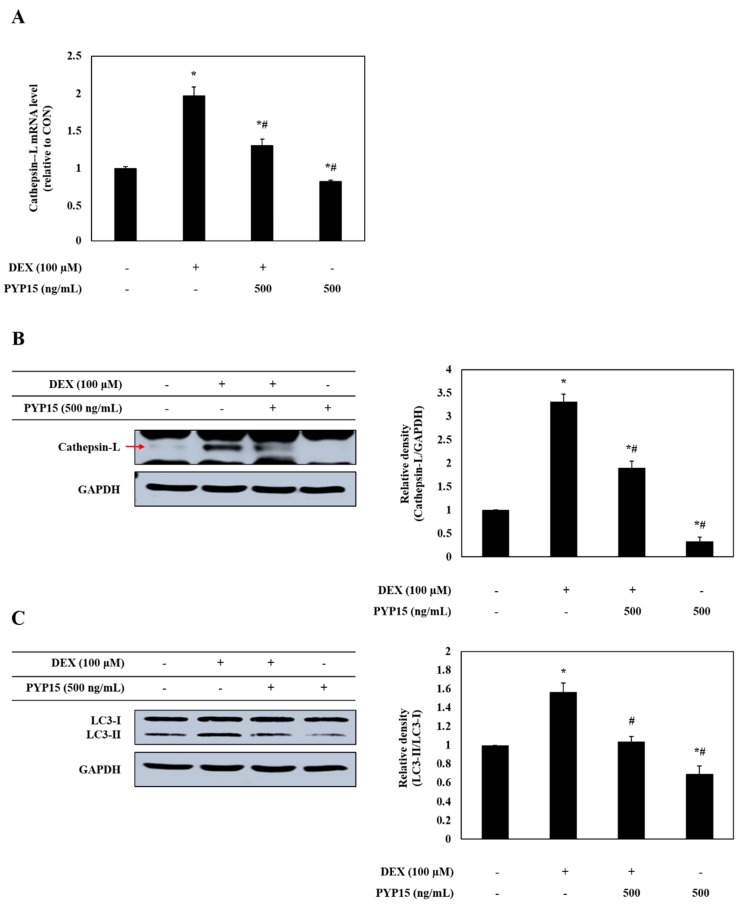
Effects of PYP15 on cathepsin-L and LC3-I/II levels in DEX-stimulated C2C12 myotubes. C2C12 myotubes were treated for 24 h with 100 μM DEX in the absence or presence of 500 ng/mL PYP15. (**A**) The mRNA levels for cathepsin-L were assessed as described in Section 4. (**B**) The protein levels for cathepsin-L and (**C**) LC3-I/II were assessed as described in Section 4. GAPDH was the loading control. The values are the mean ± SD of three independent experiments. * P < 0.05 vs. corresponding control; *# P* < 0.05 vs. corresponding only DEX treatment.

The effects of PYP15 on the expression of cathepsin-L and autophagy-related genes in DEX-treated C2C12 myotubes were assessed by real-time polymerase chain reaction (PCR) and Western blot analysis. As shown in Figure 8A,B, the mRNA and protein expression levels of cathepsin-L were significantly increased in DEX-stimulated C2C12 myotubes (*P* < 0.05). However, the DEX-induced upregulation of cathepsin-L was attenuated by 500 ng/mL PYP15 treatment. In addition, DEX treatment increased the conversion of LC3-I to LC3-II, which was attenuated by PYP15 treatment (Figure 8C).

### 2.8. Analysis of Myotube Atrophy Marker Genes after Akt siRNA Transfection

**Figure 9:**
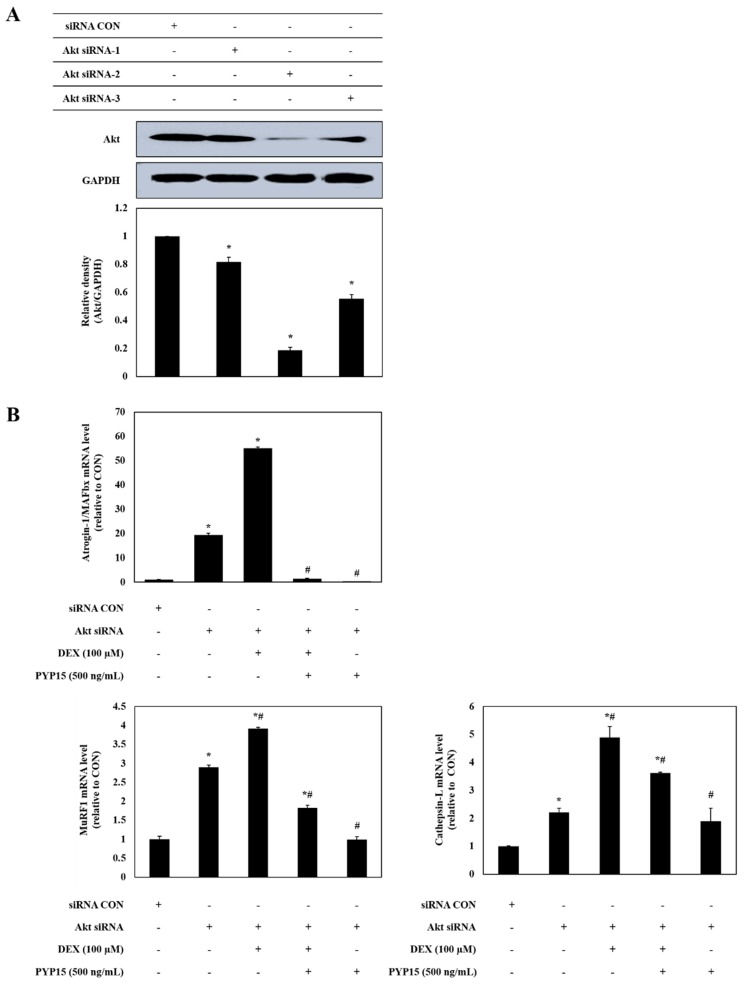

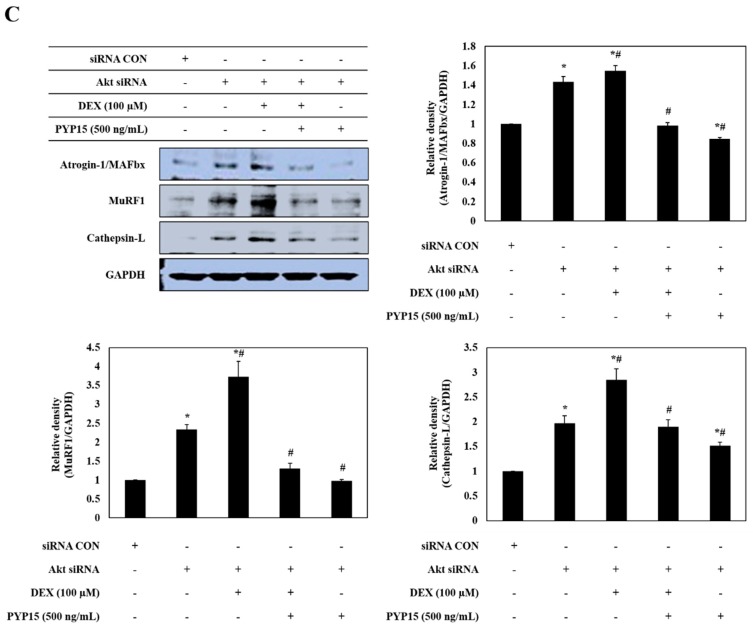
Effects of PYP15 on the levels of ubiquitin-E3 ligases following DEX-induced myotube atrophy in transfected C2C12 myotubes. (**A**) Changes in Akt protein levels by Akt knockdown were measured as described in Section 4. (**B**,**C**) The mRNA and protein levels of atrogin-1/MAFbx, MuRF1, and cathepsin-L in the five treatment groups were measured as described in Section 4. GAPDH was the loading control. The values are the mean ± SD of three independent experiments. * P < 0.05 vs. corresponding control; *# P* < 0.05 vs. corresponding only Akt siRNA treatment.

We investigated the expression of E3 ubiquitin ligases after knocking down Akt gene expression to clarify whether Akt plays a role in the PYP15-mediated inhibition of E3 ubiquitin ligases in C2C12 myotubes. As shown in Figure 9A, the protein level of Akt was markedly reduced after treatment with Akt siRNA2, in comparison with the universal negative control siRNA. These results confirmed that C2C12 myotubes were successfully transfected with Akt siRNA2. Therefore, Akt siRNA2 was used in subsequent experiments. Following this, we transfected cultured myotubes with Akt siRNA2 to test whether the effects of PYP15 on the DEX-induced upregulation of atrogin-1/MAFbx, MuRF1, and cathepsin-L expression are inhibited after Akt knockdown. Transfection of C2C12 myotubes with Akt siRNA2 significantly increased the mRNA and protein levels of atrogin-1/MAFbx, MuRF1, and cathepsin-L, and these increases in expression were attenuated by PYP15 treatment (Figure 9B,C). These results demonstrate that PYP15 prevents myotube atrophy by blocking proteolytic systems through Akt activation.

## 3. Discussion

In this study, we used an in vitro model to investigate the protective role of PYP15, and the mechanisms of its anti-atrophic effects, against DEX-induced muscle atrophy. DEX induces atrophy in skeletal muscle by decreasing the protein synthesis rate and increasing the protein degradation rate [39]. The inhibitory effect of DEX on muscle protein synthesis is mainly induced by inhibition of the IGF-I signaling pathway, which is an anabolic growth factor [40]. Previous studies have demonstrated that IGF-I is sufficient to induce skeletal muscle hypertrophy [41,42]. The inverse of muscle atrophy is muscle hypertrophy, defined as an increase in muscle mass resulting from an increase in size, as opposed to by an increase in the number of muscle fibers [28,43]. The effects of IGF-I are mainly mediated by IGF-IR, which exhibits tyrosine kinase activity and signals via adaptor proteins, such as IRS-1 [44]. In this study, the DEX-induced reductions in IGF-IR and IRS-1 phosphorylation were ameliorated by treatment with PYP15 (Figure 3). These results suggest that PYP15 could protect C2C12 myotubes from DEX-induced myotube atrophy through the activation of IGF-I signaling. The downstream signaling mechanisms induced by IGF-I required for muscle hypertrophy remain controversial, but many studies have focused on the Akt-mTOR pathway downstream of IGF-I, demonstrating that Akt is activated by IGF-I [28,45]. In our results, DEX inhibited the phosphorylation of Akt and mTOR, but treatment with PYP15 increased this phosphorylation (Figure 4A). mTOR interacts with several proteins to form two distinct multiprotein complexes, mTORC1 and mTORC2. The inhibition of protein synthesis by DEX is known to be mainly due to inhibition of mTORC1 [40]. This inhibition of mTORC1-signaling by DEX is induced by the transcriptional stimulation of REDD1 and KLF-15, which are inhibitors of mTORC1 signaling [40]. PYP15 treatment activated Raptor expression in DEX-induced C2C12 myotubes and reduced the DEX-induced expression of REDD1 and KLF15 (Figure 4). These results suggest that PYP15 inhibits muscle atrophy through activation of the mTORC1 signaling pathway in DEX-stimulated C2C12 myotubes. Activation of the Akt-mTORC1 signaling pathway stimulates protein synthesis by increasing protein translation through the activation of p70S6K and inhibition of 4E-BP1 [28]. In our study, PYP15 treatment increased the phosphorylation of p70S6K, S6, and 4E-BP1 as well as the expression of eIF4E (Figure 5). Interestingly, C2C12 myotubes treated with PYP15 alone exhibited marked upregulation of the IGF-I-Akt-mTORC1 signaling pathway compared with untreated control cells. Taken together, these results demonstrate that PYP15 could protect C2C12 myotubes from DEX-induced myotube atrophy by inducing muscle hypertrophy through the activation of Akt-mTORC1 pathway via the activation of IGF-I signaling.

DEX-induced myotube atrophy is mediated by FoxO transcription factors [18]. In addition to stimulating protein synthesis pathways, Akt activation inhibits proteolytic systems by inducing phosphorylation of the downstream target FoxO transcription factors, thus blocking nuclear translocation [17]. Previous studies have shown that the reduced activity of the Akt pathway observed in the muscle atrophy model markedly increases the levels of phosphorylated FoxO in the cytoplasm as well as the nuclear expression of FoxO [46]. In addition, FoxO transgenic mice reportedly exhibit markedly reduced muscle mass, further supporting that FoxO is sufficient to stimulate muscle atrophy [47,48]. Our results demonstrate that the phosphorylation of FoxO is activated by treatment with PYP15, resulting in decreased nuclear translocation (Figure 6). These results indicate that PYP15 effectively blocks the nuclear translocation and activation of FoxO1 and FoxO3a by promoting the phosphorylation of FoxO1 and FoxO3a.

The increased expression of FoxO contributes to the activation of muscle proteolysis through the ubiquitin-proteasome system and lysosomal system [30]. The ubiquitin-proteasome system functions in processing and degrading cellular proteins, which is essential for basic cellular processes, such as differentiation, proliferation, and immune and inflammatory responses [49,50]. The degradation of a target protein by the ubiquitin-proteasome system is labeled by the covalent bonds of multiple ubiquitin molecules, comprising 76 amino acids; the proteins are then degraded by proteolytic enzymes [51]. Previous studies have shown that the ubiquitin-proteasome pathway is downregulated by the IGF-I-mediated Akt-FoxO signaling pathway [31] and that the inhibition of proteasomal activity markedly inhibits muscle proteolysis in muscle atrophy [52]. Our study showed that the DEX-stimulated expression of atrogin-1/MAFbx and MuRF1 and the activity of the 20S proteasome were downregulated by PYP15 treatment (Figure 7). In addition to the ubiquitin-proteasome system, the proteolytic system found to be regulated by the Akt-FoxO signaling pathway is the autophagy-lysosomal system [53]. Previous studies have demonstrated the downregulation of the autophagy-lysosomal pathway by the Akt-FoxO signaling pathway [54]. DEX exerts its atrophic effects by activating the autophagy-lysosomal system through increased cathepsin-L muscle expression and increased conversion of LC3-I to LC3-II, which is an indicator of autophagy [34,35,36]. In this study, the DEX-induced increased expression of cathepsin-L and autophagy-related genes were downregulated by PYP15 treatment (Figure 8). We also confirmed that Akt knockdown induced the upregulation of atrogin-1/MAFbx, MuRF1, and cathepsin-L, effects that were reduced by PYP15 treatment (Figure 9). These results suggest that PYP15 protects DEX-induced myotube atrophy by blocking the ubiquitin-proteasome and autophagy-lysosomal pathways through downregulation of atrogenes activated by nuclear translocation of FoxO. Our results also suggest that the activation of IGF-I-mediated Akt signaling is essential for the regulation of muscle atrophy in proteolytic systems by PYP15.

In summary, these data provide molecular evidence that the anti-muscle atrophy effects of PYP15 are at least partially regulated by the Akt-mTORC1 and Akt-FoxO signaling pathways and reflect inhibited upregulation of the ubiquitin-proteasome and autophagy-lysosomal pathways.

## 4. Materials and Methods

### 4.1. Preparation of PYP15

PYP15 (D-P-K-G-K-Q-Q-A-I-H-V-A-P-S-F) was synthesized by Peptron (Daejeon, Korea). PYP15 was purified using the Shimadzu Prominence HPLC system (Shimadzu Corporation, Kyoto, Japan) and a Capcell Pak C18 column (column dimensions, 150 × 4.6 mm; particle size, 2.7 μM; Shiseido Corporation, Tokyo, Japan), with a gradient of 10% to 70% ACN (0% to 20% ACN for 2 min, 20% to 50% ACN for 10 min, 50% to 80% ACN for 2 min) in 0.1% trifluoroacetic acid (TFA; v/v in water), a flow rate of 1.0 mL/min, and UV detection at 220 nm, controlled via the software package Class-VP (ver. 6.14; Shimadzu Corporation, Kyoto, Japan). The molecular weight of PYP15 was determined to be 1622 Da using an HP 110 Series liquid chromatography/mass spectrometric detector (LC/MSD) [ionization mode, positive; nitrogen flow, 7 L/min; high vacuum, 1.3 × 10^−5^ torr; neb press, 40 psi; quadrupole temperature, 100 °C; flow rate, 0.4 mL/min (isocratic ACN: DW = 8:2, 0.1% (v/v) TFA/water); Agilent Technologies, Santa Clara, CA, USA)] [37].

### 4.2. Cell Culture and Differentiation

C2C12 mouse skeletal muscle cells were obtained from the American Type Culture Collection (CRL-1722; ATCC, Manassas, VA, USA). Cells were maintained in a humidified 5% CO_2_ incubator at 37 °C in Dulbecco’s modified Eagle’s medium (DMEM; Gibco, Thermo Fisher Scientific, Waltham, MA, USA) supplemented with 10% fetal bovine serum (FBS; Gibco, Thermo Fisher Scientific, Waltham, MA, USA), 100 U/mL penicillin (Gibco, Thermo Fisher Scientific, Waltham, MA, USA), and 100 mg/mL streptomycin (Gibco, Thermo Fisher Scientific, Waltham, MA, USA). C2C12 myoblasts were grown to 70% to 80% confluence in culture dishes (100 mm) at 37 °C, then trypsinized and seeded (4 × 10^4^ cells/well) into six-well culture plates for experiments. Cells were grown to 70% to 80% confluence in DMEM supplemented with 10% FBS at 37 °C for 24 h, at which time the medium was replaced with DMEM containing 2% FBS to induce differentiation into myotubes; the medium was replaced every 2 days. Cells were allowed to differentiate for 6 days, at which point 90% of the cells had fused into myotubes [37].

### 4.3. Treatment with DEX and PYP15

Following 6 days of differentiation, C2C12 myotubes were subdivided into four groups: The control group, in which cells were incubated in serum-free medium (SFM; DMEM containing 100 U/mL penicillin and 100 mg/mL streptomycin); the DEX group, in which cells were treated with 100 μM DEX (Sigma-Aldrich, St. Louis, MO, USA); the DEX + PYP15 group, in which cells were treated with 100 μM DEX and 500 ng/mL PYP15; and the PYP15 group, in which cells were treated with 500 ng/mL PYP15. All groups were incubated in SFM at 37 °C for 24 h prior to harvesting cells for experiments. The concentrations of DEX and PYP15 used here were based on previous studies [37].

### 4.4. MTS Assay

Cell viability was measured using the CellTiter 96 Aqueous Non-Radioactive Cell Proliferation Assay (Promega Corporation, Madison, WI, USA), which is based on the formation of a formazan product from tetrazolium compound MTS [(3-4,5-dimethylthiazol-2-yl)-5-(3-carboxymethoxyphenyl)-2-(4-sulfonyl)-2H-tetrazolium)]. Briefly, cells (1.5 × 10^4^ cells/well) were seeded into 96-well plates in 100 μL DMEM supplemented with 10% FBS and were allowed to attach at 37 °C for 24 h. After differentiation, the cells were incubated with 100 μM DEX and 500 ng/mL PYP15 for 24 h at 37 °C. MTS solution (10 μL) was added and the cells were incubated at 37 °C for 30 min. The absorbance at 490 nm was measured using a Gen5 ELISA (Bio-Tek, Houston, TX, USA) [37]. Experiments were performed in triplicate.

### 4.5. Real-Time PCR

The mRNA expression levels of specific genes were evaluated using real-time PCR. Total RNA was isolated from C2C12 myotubes using TRIzol reagent (Invitrogen Life Technologies, Carlsbad, CA, USA). The resulting RNA was evaluated by measuring the absorbance at 260 and 280 nm to determine the RNA concentration and purity, respectively. A RevoScript Reverse Transcriptase PreMix Kit (Intron Biotechnology Co., Ltd., Seongnam, Korea) was used to prepare cDNA according to the manufacturer’s instructions, and the samples were stored at −50 °C. Real-time PCR was conducted in 20-μL reactions using the TOPreal qPCR 2X preMIX (Enzynomics, Inc., Daejeon, Korea) and the Illumina Eco real-time PCR system (Illumina, Inc., Hayward, CA, USA). All mRNA levels were normalized using glyceraldehyde-3-phosphate dehydrogenase (GAPDH) as an internal control [37]. The primers used for amplification are shown in Table 1.

### 4.6. Preparation of Total Cell Lysates

Cell were allowed to differentiate for 6 days at 37 °C, followed by incubation at 37 °C for 24 h in either SFM (control group) or SFM containing 100 μM DEX (DEX group), 100 μM DEX + 500 ng/mL PYP15 (DEX + PYP15 group), or 500 ng/mL PYP15 (PYP15 group). Cells were washed twice with PBS (Gibco, Thermo Fisher Scientific) and lysed with extraction buffer [1% NP-40, 0.25% sodium deoxycholate, 1 mM ethylene glycol-bis (β-aminoethyl ether)-*N,N,N’N’*-tetraacetic acid, 150 mM NaCl, and 50 mM Tris-HCl, pH 7.5] containing protease inhibitors (1 mg/mL aprotinin, 1 mg/mL leupeptin, 1 mg/mL pepstatin A, 200 mM Na_3_VO_4_, 500 mM NaF, and 100 mM PMSF) on ice. After incubation for 30 min at 4 °C, the extracts were centrifuged at 16,000 × *g* for 10 min at 4 °C, and protein levels were quantified using a bicinchoninic acid (BCA) protein assay kit (Pierce, Thermo Fisher Scientific) according to the manufacturer’s instructions. The supernatant was then used in Western blot analysis [37].

### 4.7. Preparation of Cytosolic and Nuclear Extracts

Cells were treated and harvested as described above, lysed with hypotonic lysis buffer (25 mM HEPES, pH 7.5, 5 mM EDTA, 5 mM MgCl_2_, and 5 mM DTT) containing protease inhibitors (1 mg/mL aprotinin, 1 mg/mL leupeptin, 1 mg/mL pepstatin A, 200 mM Na_3_VO_4_, 500 mM NaF, and 100 mM PMSF), and incubated for 15 min on ice. Cells were further lysed by adding 2.5% NP-40. After 10 min, nuclei were collected by centrifugation at 7500 × *g* for 15 min at 4 °C. The supernatant (cytosolic fraction) was immediately transferred to clean pre-chilled tubes. The insoluble (pellet) fraction is the nuclear fraction. The nuclear fraction was resuspended in cell extraction buffer (10 mM HEPES pH 7.9, 100 mM NaCl, 1.5 mM MgCl_2_, 0.1 mM EDTA, and 0.2 mM DTT) containing protease inhibitors (1 mg/mL aprotinin, 1 mg/mL leupeptin, 1 mg/mL pepstatin A, 200 mM Na_3_VO_4_, 500 mM NaF, and 100 mM PMSF). The samples were placed on ice and vortexed for 15 s every 10 min for a total of 40 min. Extracts were centrifuged at 16,000 × *g* for 10 min, and protein levels were determined using a BCA protein assay kit (Pierce, Thermo Fisher Scientific, Waltham, MA, USA) according to the manufacturer’s instructions. Both fractions were then used in Western blot analysis [37].

### 4.8. Western Blot Analysis

Equal amounts of proteins (40 μg) were separated by 6% to 12.5% sodium dodecyl sulfate-polyacrylamide gel electrophoresis (SDS-PAGE) and transferred to a polyvinylidene fluoride membrane (Millipore, Bedford, MA, USA). The membrane was blocked at room temperature with 1% bovine serum albumin (BSA) in TBS-T (10 mM Tris-HCl, 150 mM NaCl, and 0.1% Tween-20) and then incubated with primary antibodies (Table 2). The secondary antibodies (diluted 1:10,000 to 1:20,000) were horseradish peroxidase-conjugated anti-rabbit IgG (7074S; Cell Signaling Technology, Inc., Beverly, MA, USA), donkey anti-goat IgG (A50-101P; Bethyl Laboratories, Inc., Montgomery, TX, USA), or goat anti-mouse IgG (sc-2031; Santa Cruz Biotechnology, Inc., Santa Cruz, CA, USA). Signals were detected using an enhanced chemiluminescence Western blot analysis kit (Thermo Fisher Scientific). Experiments were performed in triplicate and densitometry analysis was performed using Multi-Gauge software version 3.0 (Fujifilm Life Science, Tokyo, Japan) [37].

### 4.9. Akt Small Interfering RNA (siRNA) Transfection

For gene silencing, three different siRNA oligonucleotides (predesigned and synthesized by Bioneer, Daejeon, Korea) targeting Akt (GenBank accession No. NM_009652.2) were transfected into differentiated C2C12 myotubes using Lipofectamine RNAiMAX transfection reagent (Invitrogen Life Technologies) according to the manufacturer’s instructions. The siRNA sequences are listed in Table 3. Negative controls were employed to evaluate siRNA specificity and for siRNA optimization. Briefly, siRNA (50 μM) and Lipofectamine RNAiMAX were separately diluted in Opti-MEM (Invitrogen Life Technologies) and then combined. The mixture was incubated for 20 min at room temperature and added to the cells for 24 h at 37 °C. After 24 h, 4% FBS and antibiotic/antimycotic-free DMEM were added to the cells, resulting in a final FBS concentration of 2%. The cells were further incubated for 24 h in a CO_2_ incubator to silence Akt expression. The efficiency of Akt siRNA silencing was assessed by measuring Akt protein levels by Western blotting. After siRNA transfection, myotubes were exposed to 100 μM DEX and 500 ng/mL PYP15 for 24 h.

### 4.10. 20S Proteasome Activity Assay

The chymotrypsin-like activity of the 20S proteasome was measured as changes in the fluorescence of 7-amino-4-methylcoumarin (AMC) conjugated to the chymotrypsin peptide substrate LLVY, using a 20S proteasome activity assay kit (Chemicon, Temecula, CA, USA). Briefly, cells were suspended in RIPA lysis buffer (50 mM Tris-HCl, pH 7.5, 150 mM sodium chloride, 0.5% sodium deoxycholate, 1% Triton X-100, 0.1% SDS, and 2 mM EDTA) containing protease inhibitors (1 mg/mL aprotinin, 1 mg/mL leupeptin, 1 mg/mL pepstatin A, 200 mM Na_3_VO_4_, 500 mM NaF, and 100 mM PMSF) and centrifuged at 16,000 × *g* for 10 min at 4 °C. The protein concentration in supernatants was determined using the BCA protein assay kit (Pierce). The cell lysates were incubated for 90 min at 37 °C with a labeled substrate, Leu-Leu-Val-Tyr (LLVY)-AMC, and the cleavage activity was monitored by detecting the free fluorophore AMC using a fluorescence plate reader (Gen5 ELISA, Bio-Tek, Winooski, VT, USA).

### 4.11. Statistical Analysis

Mean values were compared by analysis of variance using SPSS software (ver. 10.0; SPSS Inc., Chicago, IL, USA). The values are presented as the mean ± SD. Different letters indicate significant differences among groups according to Duncan’s multiple-range test [37].

## Figures and Tables

**Table 1:** Oligonucleotide primer sequences used in real-time PCR.

Gene	Accession No.	Sequence (5′–3′)	Amplicon Size (bp)
Atrogin-1/MAFbx	NM_026346.3	F: ATGCACACTGGTGCAGAGAG R: TGTAAGCACACAGGCAGGTC	168
Cathepsin-L	M20495.1	F: GACCGGGACAACCACTGTG R: CCCATCAATTCACGACAGGAT	61
GAPDH	NM_008084.3	F: ACTCCACTCACGGCAAATTCA R: CGCTCCTGGAAGATGGTGAT	91
KLF-15	NM_001355668.1	F: CGAGAAGCCCTTTGCCTGCA R: ATCGCCGGTGCCTTGACAAC	70
MuRF1	DQ_229108.1	F: CGAGAAGCCCTTTGCCTGCA R: GTGCCGGTCCATGATCACTT	59
REDD1	NM_029083.2	F: TGGTGCCCACCTTTCAGTTG R: GTCAGGGACTGGCTGTAACC	121

**Table 2:** Primary antibodies used in Western blot analysis.

Antibody	Manufacturer and Catalog No.	Species of Origin	Dilution Rate
4E-BP1	Santa Cruz Biotechnology: sc-9977	Mouse	1:1000
Akt	Santa Cruz Biotechnology: sc-8312	Rabbit	1:1000
Atrogin-1/MAFbx	Santa Cruz Biotechnology: sc-27645	Goat	1:2000
β-actin	Santa Cruz Biotechnology: sc-47778	Mouse	1:1000
Cathepsin-L	Santa Cruz Biotechnology: sc-6498	Goat	1:1000
eIF4E	Santa Cruz Biotechnology: sc-514875	Mouse	1:1000
FoxO1	Santa Cruz Biotechnology: sc-374427	Mouse	1:500
FoxO3a	Santa Cruz Biotechnology: sc-9813	Goat	1:1000
GAPDH	Santa Cruz Biotechnology: sc-25778	Rabbit	1:1000
IGF-IR	Santa Cruz Biotechnology: sc-713	Rabbit	1:1000
IRS-1	Santa Cruz Biotechnology: sc-560	Rabbit	1:1000
KLF-15	Santa Cruz Biotechnology: sc-27165	Mouse	1:1000
Lamin B	Santa Cruz Biotechnology: sc-377000	Mouse	1:1000
LC3-I/II	Cell Signaling: #4108S	Rabbit	1:1000
mTOR	Santa Cruz Biotechnology: sc-8319	Rabbit	1:1000
MuRF1	Santa Cruz Biotechnology: sc-27642	Goat	1:2000
p-4E-BP1	Santa Cruz Biotechnology: sc-293124	Mouse	1:1000
p70S6K	Santa Cruz Biotechnology: sc-8418	Mouse	1:1000
p-Akt	Santa Cruz Biotechnology: sc-135650	Mouse	1:500
p-FoxO1	Cell Signaling: #9461S	Rabbit	1:500
p-FoxO3a	Cell Signaling: #9466S	Rabbit	1:1000
p-IGF-IR	Santa Cruz Biotechnology: sc-101703	Rabbit	1:1000
p-IRS-1	Santa Cruz Biotechnology: sc-17200	Goat	1:1000
p-mTOR	Santa Cruz Biotechnology: sc-293132	Mouse	1:1000
p-p70S6K	Santa Cruz Biotechnology: sc-8416	Mouse	1:1000
p-S6	Santa Cruz Biotechnology: sc-293144	Mouse	1:1000
Raptor	Santa Cruz Biotechnology: sc-81537	Mouse	1:1000
REDD1	Santa Cruz Biotechnology: sc-376671	Mouse	1:1000
Rheb	Santa Cruz Biotechnology: sc-271509	Mouse	1:1000
Rictor	Santa Cruz Biotechnology: sc-81538	Mouse	1:1000
S6	Santa Cruz Biotechnology: sc-74459	Mouse	1:1000

**Table 3:** Small interfering RNA (siRNA) sequences used for Akt knockdown.

Gene	Accession No.	Sequence (5′–3′)
Akt siRNA1	NM_009652.2	F: CUCAAGUGAGGUUGACAGA R: UCUGUCAACCUCACUUGAG
Akt siRNA2	NM_009652.2	F: CCACGGAUACCAUGAACGA R: UCGUUCAUGGUAUCCGUGG
Akt siRNA3	NM_009652.2	F: GACGAUGGACUUCCGAUCA R: UGAUCGGAAGUCCAUCGUC
